# Deciphering Transcription-Translation-Folding (TX-TL-FD) for Enhancing Cutinase Production in T7 System and Genetic Chaperone-Equipped *Escherichia coli* Strains

**DOI:** 10.1021/acssynbio.5c00245

**Published:** 2025-05-07

**Authors:** Chuan-Chieh Hsiang, I-Son Ng

**Affiliations:** Department of Chemical Engineering, National Cheng Kung University, Tainan 701, Taiwan

**Keywords:** T7 RNA polymerase, transcription, translation initiation region, chaperone, cutinase, *Escherichia coli*

## Abstract

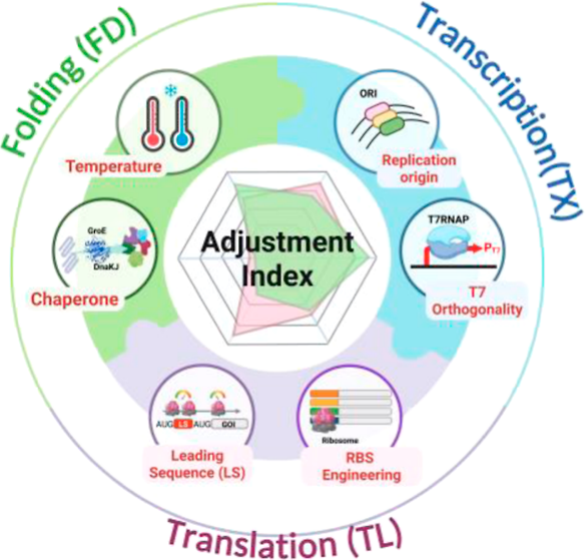

T7 RNA polymerase (T7RNAP), orthogonal to the T7 promoter, is a powerful tool in engineered *Escherichia coli* that enables the production of many different harsh enzymes. Still, it requires precise control, particularly when expressing toxic proteins. The optimized strategy for the interconnected processes of transcription (TX), translation (TL), and protein folding (FD) in the T7 system is still not well understood. Therefore, we developed a quantitative adjustment index (AI) to evaluate all regulatory factors within the “tri-synergistic TX-TL-FD” pathway to obtain high-level production of leaf-branch compost cutinase mutant (ICCM), an enzyme challenging to express in soluble form. Among six *E. coli* chassis (BD, B7G, BKJ, C43, C7G, and CKJ), and considering the effect of replication origin, ribosome binding site (RBS), and chaperones, we identified T7RNAP level and translation initiation region (TIR) as the primary determinants of expression efficiency. Coordinated regulation of TX-TL proved the most effective performance, thus enhancing ICCM expression by 90%. In contrast, FD optimization through temperature modulation yielded only 10% enhancement. Notably, molecular chaperones of GroELS and DnaK/J showed benefits only after achieving optimal TX-TL balance. This hierarchical framework of trisynergistic regulation in the T7 system provides a universal strategy to express complex proteins in engineered *E. coli*.

## Introduction

The orthogonality of T7 RNA polymerase (T7RNAP) with the T7 promoter has been indispensable for initiating protein expression in genetically engineered *Escherichia coli* for decades. It also applies to various organisms, including *Bacillus subtilis*, yeast, and cyanobacteria, as well as cell-free systems, spanning both prokaryotic and eukaryotic systems.^1^ However, the high energy demands associated with the T7 system,^2−5^ particularly when expressing toxic and stress-inducing proteins, are known to hinder cell growth and cause cell lysis during cultivation.^6−10^ This implies that the T7 system in engineered cells requires more precise regulation.

Successful enzyme production depends on the coordinated interaction of transcription, translation, and folding processes (TX-TL-FD). Previous studies have employed mutated strains with reduced T7 orthogonality to lower transcriptional levels and balance the challenge of protein production. For example, Miroux and Walker isolated *E. coli* BL21(DE3) mutant derivatives, C41(DE3) and C43(DE3), showing a point mutation in the *lac*UV5 promoter to control T7RNAP, which achieved higher yields and reduced toxicity, thus is widely used for membrane protein production.^11^ On the other hand, Lemo21(DE3), a BL21(DE3) derivative, enables precise control of T7RNAP through tunable T7 lysozyme using rhamnose as inducer,^12,13^ while the Mutant56(DE3) harboring a T7RNAP mutation that weakens promoter, achieves more membrane protein yields.^14,15^

Other studies focus on improving translation efficiency but often overlook the context-dependent role of the translation initiation region (TIR), which includes the Shine-Dalgarno sequence, 5′-UTR, and a short leader sequence upstream of the N-terminal of recombinant genes, all of which influence initiation rates in prokaryotes.^16^ Shilling et al. generated two TIR libraries that boosted transcription and translation initiation by screening from 30,000 and 16 million variants to test sfGFP production.^17^ Ribosome binding sites (RBSs) affect translation efficiency and fidelity and directly influence protein abundance and quality.^18^ Therefore, a library of RBS variants with different strengths offers a powerful tool for gene expression control.^19−22^ On the other hand, chaperones of GroELS and DnaKJ play a vital role in ensuring proper protein folding (FD) and enhancing overall yield in engineered *E. coli*.^23−27^ Thus, considering the transcription, translation, and folding, annotated as the TX-TL-FD pathway, is crucial for assessing regulatory interactions and developing universal guidelines to optimize challenging protein production.

Till now, the trisynergistic TX-TL-FD regulation for harsh protein expression has yet to be fully elucidated. Recently, cutinase ICCM featuring four mutations (F243I, D238C, S283C, and Y127G) that enhance thermostability has emerged as a key enzyme for degrading polyethylene terephthalate (PET) waste using Lemo21(DE3).^28,29^ However, ICCM has poor solubility and may exhibit potential cytotoxic effects on *E. coli* when overexpressed. Therefore, we investigated the synergistic relationship within the TX-TL-FD process to improve ICCM yield by investigating regulatory aspects, developing an adjustment index for control factors such as host with different levels of T7RNAP, plasmid copy numbers, strength of RBS, cultural temperature, and chaperones to assess their significance. We proposed a highly efficient optimization strategy for the trisynergistic nature of the harsh protein expression in engineered *E. coli*.

## Results and Discussion

### Transcription (TX) Regulation in Protein Overexpression

In the host equipped with the T7 system, several regulatory factors control the efficiency of protein transcription, including the strength of T7 orthogonality, the number of active plasmids in the cell, and the termination efficiency of T7RNAP on the DNA transcript. The ways to affect the T7 orthogonality in *E. coli* were diverse and not limited to changing the T7RNAP levels and its binding affinity with the T7 promoter.^17,30^ The requirement of active plasmids in the host is also an essential concern during protein overexpression. The number of plasmids affects transcription efficiency, especially under polymerase-limited circumstances.^31,32^

**Figure 1:**
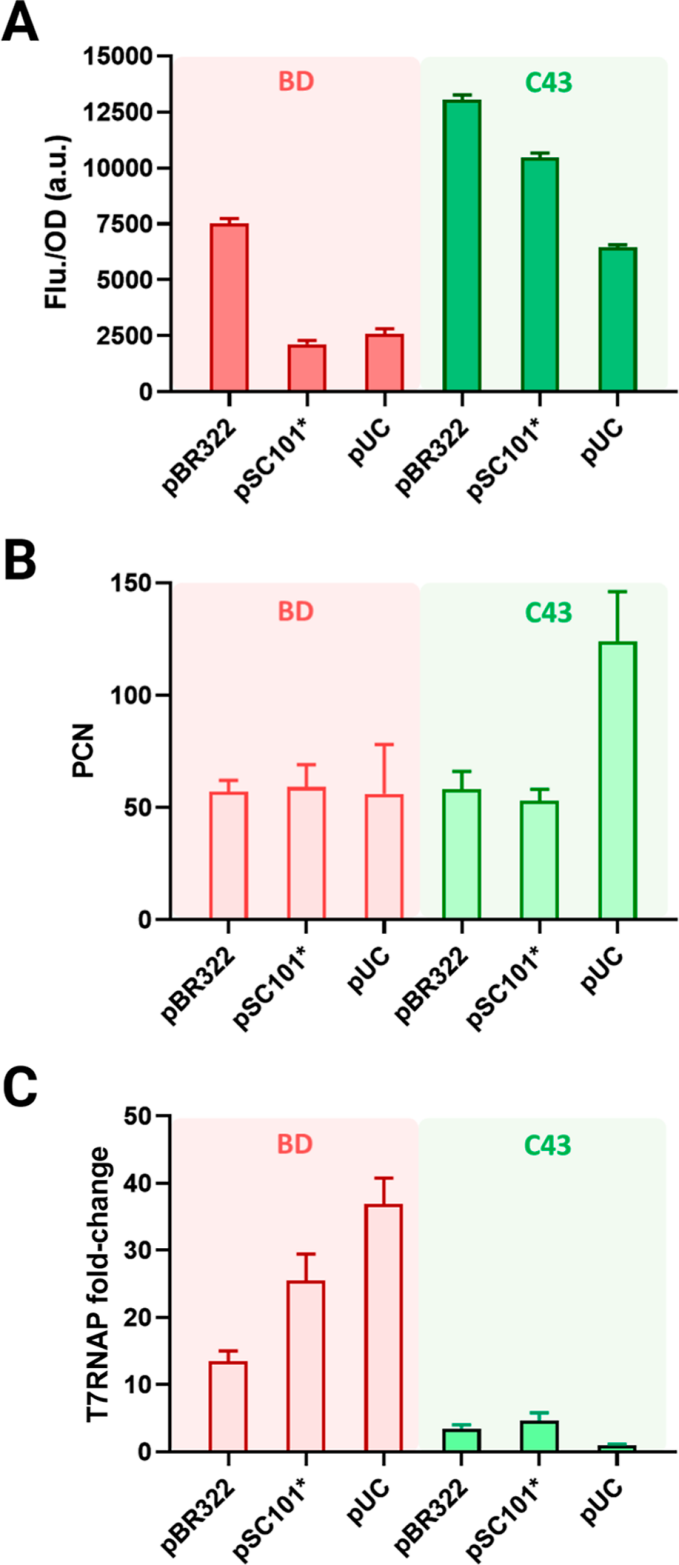
Vector’s replication origin affects ICCM expression in BD and C43 strains. (A) Specific fluorescence, (B) plasmid copy number (PCN), (C) relative T7RNAP level in BD and C43, respectively. The lowest T7RNAP level in C43 harboring pUC vector is defined as 1. All cells were induced with 0.1 mM IPTG at 37 °C. Samples for PCN and T7RNAP in the measurements were collected after postinduction at 5 h. Error bars represent three independent standard deviations (SD) (*n* = 3).

To begin with, the replication origin of pET vector was replaced from pBR322 to pSC101*(i.e., 2-point mutations on this origin of replication) and pUC, while the two new vectors of pSCKI-T7S-ICCM-sfGFP and pSUI-T7S-ICCM-sfGFP were constructed. Besides, *E. coli* C43(DE3) was known for maximizing the expression of hard-to-express proteins (i.e., membrane proteins) with lower T7RNAP levels during protein overexpression. Therefore, *E. coli* BL21(DE3) and C43(DE3), annotated in short as BD and C43, were applied for comparison using vectors with different replication origins (pBR322, pSC101*, and pUC) as shown in Figure 1. The fusion protein ICCM-sfGFP was expressed after 16 h induction and quantified via specific fluorescence (a.u./OD_600_). It showed that pBR322 yielded 7516 au/OD_600_ in BD, while pSC101* and pUC vectors in BD showed lower fluorescence. In the C43, plasmids of pBR322, pSC101*, and pUC achieved 13,031, 10,456, and 6447 au/OD_600_, which were 2-fold compared to that of BD strains (Figure 1A).

When we measured the plasmid copy number (PCN) and mRNA level of T7RNAP at 6 h after postinduction, all strains had comparable PCN (i.e., 55), except for pUC *ori* in C43, reaching PCN of 124 (Figure 1B; Table 1). Moreover, the T7RNAP level of all BD strains was substantially higher than that of C43 strains. When defining T7RNAP level in C43 harbored pUC vector as the control, the pBR322, pSC101, and pUC origins exhibited 13-, 25-, and 36-fold increases in BD (Figure 1C). It was observed that the intracellular T7RNAP level in BD after IPTG induction varied depending on the replication origins. At the same time, all vectors in C43 maintained a limited but stable T7RNAP level. The trade-off between ICCM-sfGFP expression and T7RNAP highlights the importance of transcription efficiency for optimal protein production. On the other hand, plasmid stability should be considered in high-copy systems and long-term cultures, whereas low-copy plasmids tend to be more stable in short-term cultures within 24 h.

**Table 1:** Quantification of Plasmid Copy Number and Relative Expression Level of T7RNAP from 2 Strains Harboring 3 Different Plasmids

strains	origin of plasmid	PCN^a^	relative T7RNAP^b^
BD	pBR322	57 ± 5	13.48 ± 1.56
BD	pSC101	59 ± 10	25.47 ± 3.96
BD	pUC	56 ± 22	26.92 ± 3.84
C43	pBR322	58 ± 8	3.46 ± 0.55
C43	pSC101	53 ± 5	4.66 ± 1.77
C43	pUC	124 ± 22	1.00 ± 0.16

a The strains were cultured in LB medium and induced by 0.1 mM IPTG. The PCN was quantified by qPCR.

b The relative expression of T7RNAP was quantified by qRT-PCR.

### Translation (TL) Regulation in Protein Overexpression

The translation step is vital in protein synthesis, as it directly determines the protein’s amino acid sequence and structure, affecting its function and stability. This process includes initiation, elongation, and termination, where the ribosome reads mRNA, assembles amino acids into a chain, and releases the completed protein at the stop codon. The translation initiation region (TIR), an mRNA sequence near the start codon, significantly influences initiation rates and includes elements such as the Shine-Dalgarno sequence,^33−35^ the 5′-UTR,^36,37^ or a short leader sequence preceding the gene of interest in prokaryotes. The TIR’s sequence, length, and secondary structure affect ribosome binding and translation efficiency, thus influencing protein expression levels.

**Figure 2:**
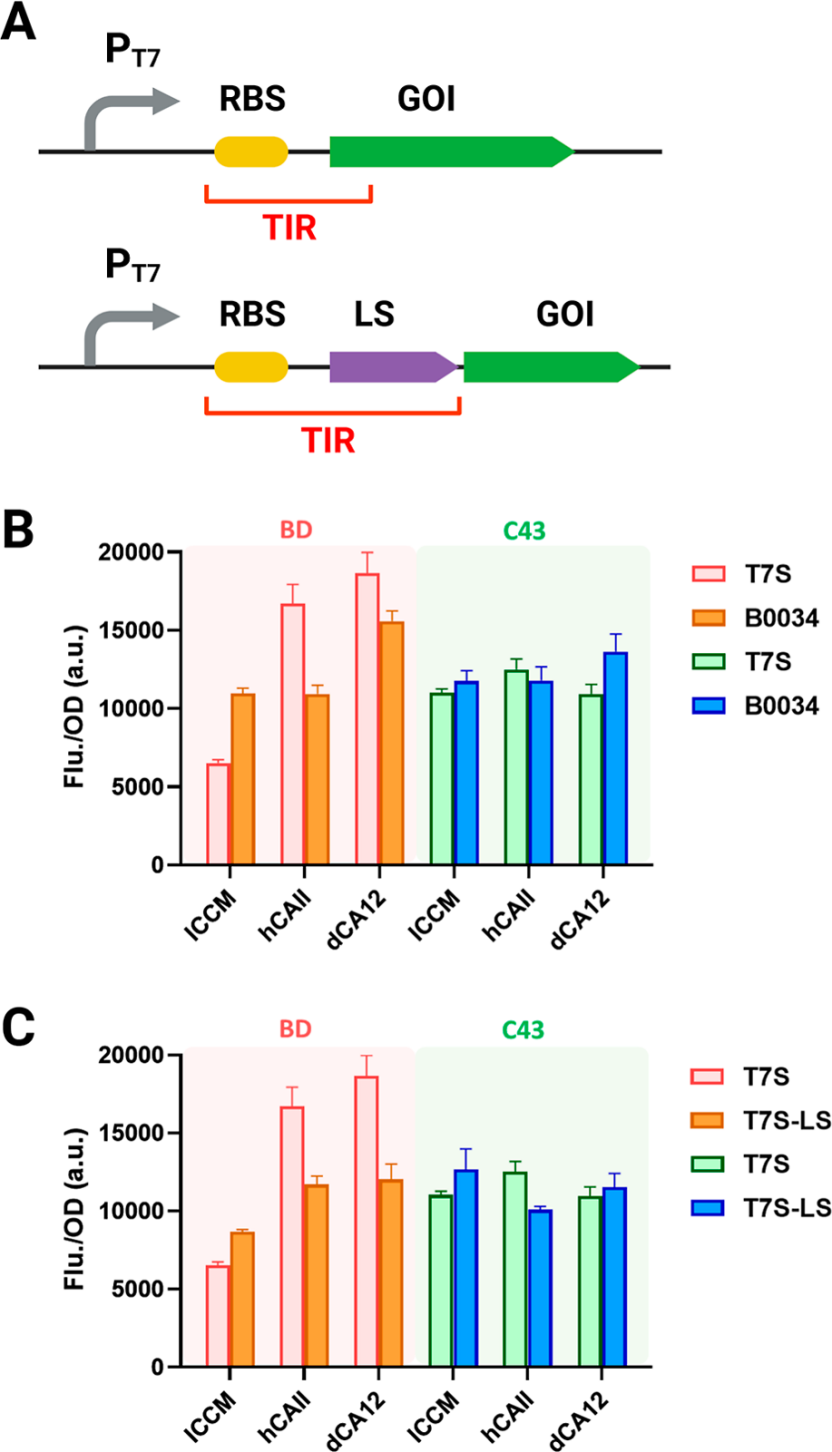
Translation initiation region (TIR) affects ICCM expression in BD and C43 strains. (A) The scheme of translation initiation efficiency through ribosome binding sites and the addition of leader sequences in TIR. (B) RBS and (C) leader sequence (LS) effects on fusion proteins ICCM-sfGFP, hCAII-sfGFP, and dCA-sfGFP. Error bars represent three independent standard deviations (SD) (*n* = 3).

The effect of the ribosome binding site (RBS), sequences of various genes of interest (GOI), and the leader sequence (LS) in the front of the GOI on the translation initiation region (TIR) and expression efficiency were further investigated (Figure 2A). It was observed that changing the original RBS following the T7 promoter (T7S) into synthetic B0034 RBS increased ICCM-sfGFP production twice in the BD strain (Figure 2B, BD) and reached a specific fluorescence of 11,000 au/OD_600_. Additionally, two other genes of interest, hCAII-sfGFP and dCA12-sfGFP, were expressed using the same vector in the BD strain and produced specific fluorescence of 16,700 and 18,660 au/OD_600_, respectively. Contrary to expectations, using the B0034 RBS reduced the expression levels of both hCAII-sfGFP and dCA12-sfGFP in BD strains. The B0034 RBS, when used with the T7 promoter, was previously reported to have a weaker translation efficiency than the T7S RBS.^38^ Therefore, the fluorescence reduction of hCAII-sfGFP and dCA12-sfGFP in BD strains aligned with the expected decrease in RBS strength and translation efficiency within the TIR. The cutinase ICCM, unlike hCAII and dCA12, is known to be challenging to express in BD strains and requires precise regulation of T7 orthogonality for optimal overexpression.^28,29^

Consequently, the fluorescence intensity of B0034-ICCM-sfGFP improved when translation efficiency in the TIR was reduced according to the results for B0034-hCAII-sfGFP and B0034-dCA12-sfGFP. In C43 strains, B0034-hCAII-sfGFP and B0034-dCA12-sfGFP expressions were consistently repressed, stabilizing fluorescence levels around 10,000 au/OD_600_ as B0034-ICCM-sfGFP (Figure 2B, C43). Changes in RBS, which reduced translation efficiency within the TIR, had a minimal impact on the expression levels across all three proteins, regardless of their expression difficulty. When T7RNAP levels were minimized, this suggested that transcription is the primary factor in controlling the protein expression process, setting the limit for expression potential.

The addition of a leader sequence (LS) has been shown to rescue the expression of challenging fusion proteins in C43(DE3) (Figure S1). Adding an LS after the T7S RBS increased ICCM-sfGFP/BD fluorescence to 8651 au/OD_600_. In contrast, LS-hCAII-sfGFP/BD and LS-dCA12-sfGFP/BD expression dropped to around 12,000 au/OD_600_ (Figure 2C). This outcome mirrors the effects of using a weaker RBS, suggesting that inserting the leader sequence functions similarly to modifying the RBS in the translation initiation region, effectively reducing the translation rate. In C43 strains, the LS-ICCM-sfGFP construct increased fluorescence to 12,688 au/OD_600_, while LS-hCAII-sfGFP and LS-dCA12-sfGFP showed similar fluorescence levels to those in BD strains (Figure 2C). This result suggested that transcriptional regulation remained dominant in C43 strains, even with the insertion of a leader sequence in the TIR.

### Temperature and Chaperone Affect the Folding (FD) Process

Culture temperature is crucial in protein folding efficiency in *E. coli* expression systems. Relatively lower temperatures improve folding by decelerating translation, allowing the polypeptide chain time to achieve the correct conformation and reducing misfolded aggregates or inclusion bodies. In contrast, higher temperatures can accelerate translation, potentially overwhelming the cellular folding machinery and leading to misfolded proteins.^39^ However, some thermally stable proteins may require higher temperatures to fold correctly. Additionally, moderate increases in temperature can trigger heat-inducible chaperones, such as DnaK and GroE, which support protein folding.^40,41^ This study examined the temperature effect on ICCM-sfGFP expression, focusing on 30 °C, 37 °C, and 42 °C as low, medium, and high induction temperatures, respectively. The P_T7_-GroELS and P_T7_-DnaKJ chaperone clusters were integrated into the chromosome of BD and C43 strains, constructing strains B7G, BKJ, C7G, and CKJ.

**Figure 3:**
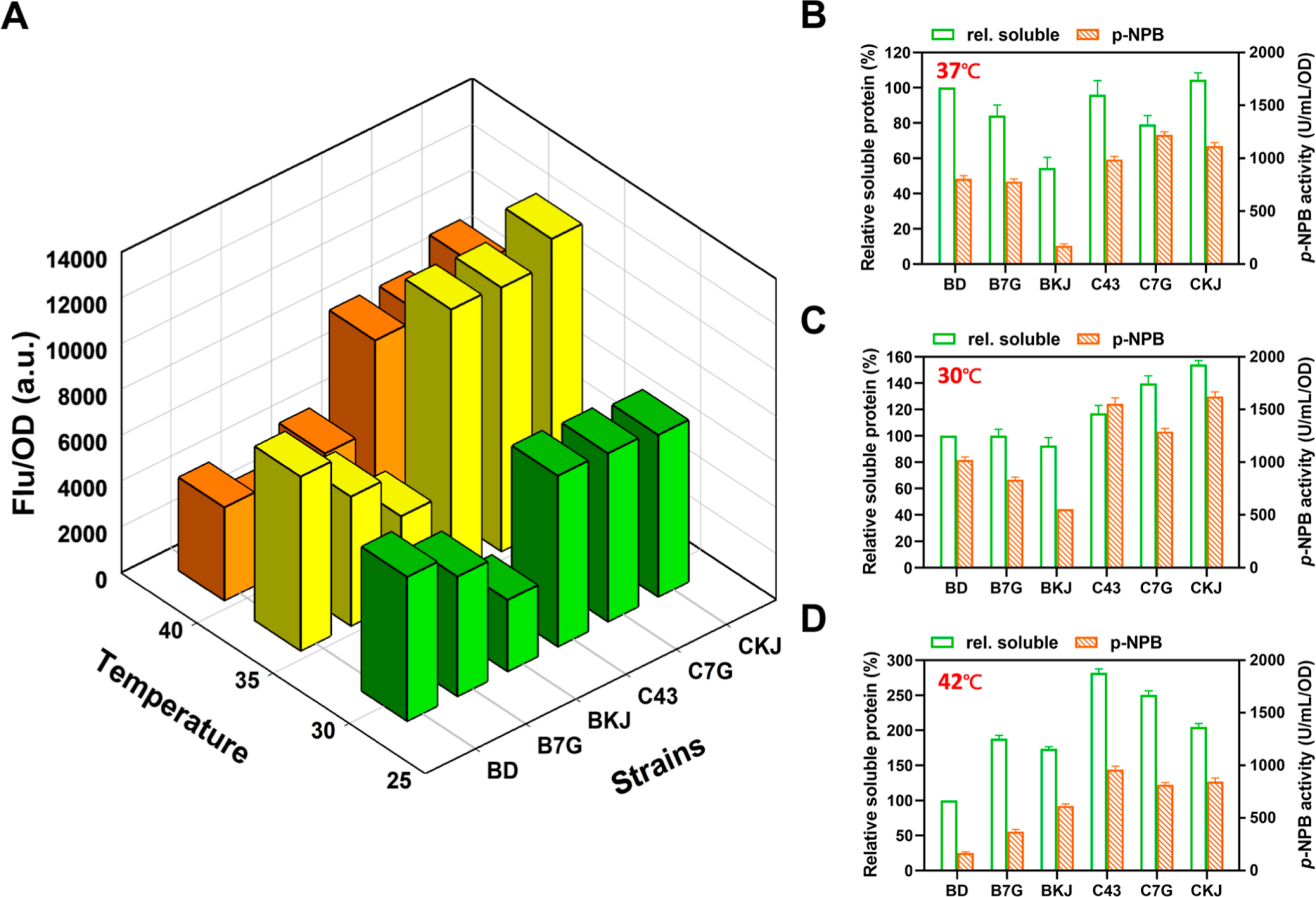
(A) The chaperone effect on the fluorescence result of ICCM-sfGFP was analyzed at 30, 37, and 42 °C. The solubility and *p*-NPB activity in BD and C43 strains at (B) 37 °C, (C) 30 °C, and (D) 42 °C. Error bars represent three independent standard deviations (SD) (*n* = 3).

In the starting BD strain, ICCM-sfGFP has the highest fluorescence at 37 °C, while B7G and BKJ strains showed poorer results. The fluorescence in C43, C7G, and CKJ strains was also affected by induction temperature. Results showed that 37 °C was optimal across all C43-based strains, while increasing the temperature to 42 °C significantly reduced the fluorescent signal (Figure 3A). The relative soluble protein yield and enzyme activity of ICCM-sfGFP at various induction temperatures were assessed using SDS-PAGE and the *p*-NPB assay (Figure 3B–D). ICCM-sfGFP in BD at 30 and 37 °C were the standard for comparing soluble protein, while GroELS and DnaKJ chaperones neither improved soluble protein nor ICCM activity (Figure 3B,C). In contrast, the relative soluble protein amount and activity were enhanced compared to the control strain ICCM-sfGFP/BD at 42 °C (Figure 3D). The impact of assisting chaperones on protein folding was particularly evident at higher induction temperatures. This suggested adjusting the induction temperature, decelerated translation, and enhanced folding more effectively than chaperone expression alone. It also showed that both protein yield and enzyme activity were influenced by induction temperature in strains with lower transcription levels.

The *p*-NPB activity of ICCM-sfGFP peaked in C43, C7G, and CKJ strains at 30 °C (Figure 3C). While C43-based strains had higher enzyme activity and protein than BD-based strains, both declined with higher temperatures (Figure 3D). As a hard-to-express protein, ICCM-sfGFP showed optimal expression at moderate temperatures in BD strains, with limited benefit from coexpressed chaperones. For C43 strains with weaker T7RNAP transcription, temperature was critical for enhancing protein expression and activity.

**Figure 4:**
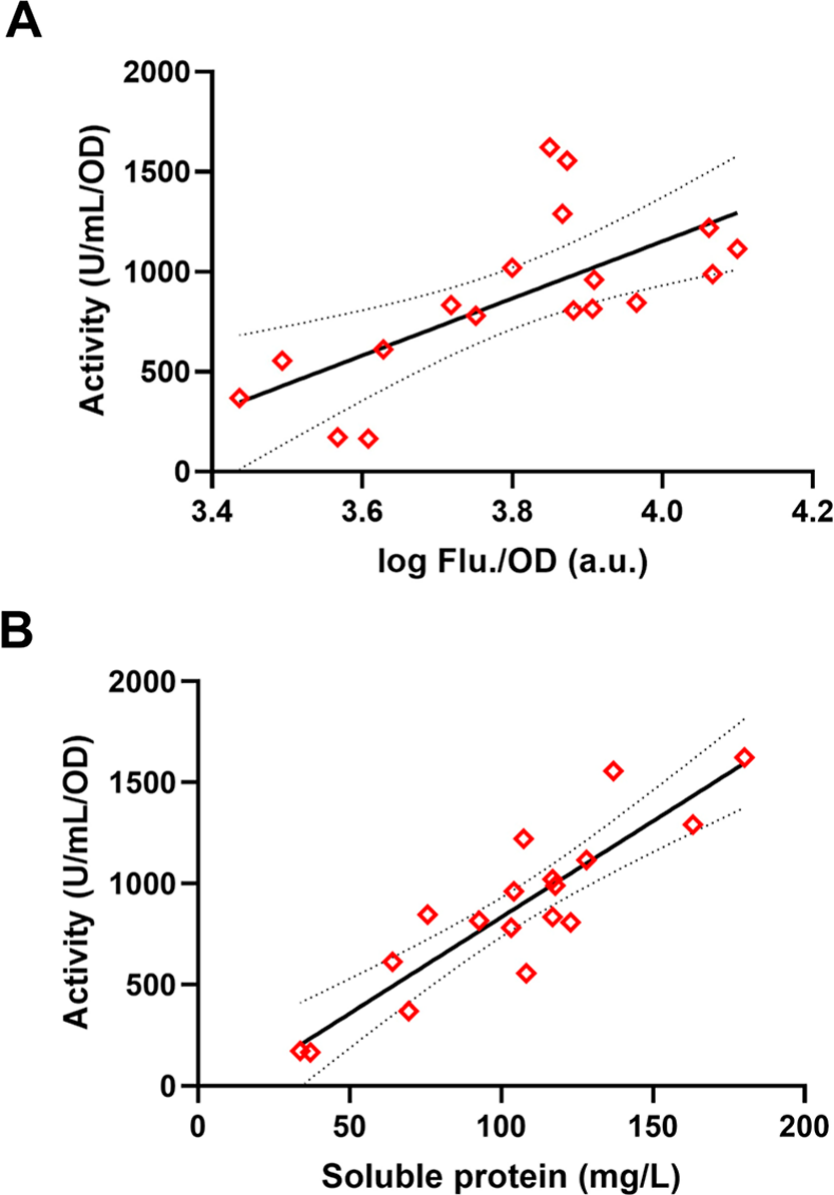
Regression relationship between *p*-NPB activity and (A) fluorescence or (B) soluble protein was investigated. Error bars represent three independent standard deviations (SD) (*n* = 3).

We employed fluorescence as a primary parameter to quantify expression levels due to its ease of normalization. Interestingly, analysis of fluorescence (a.u./OD_600_), activity, and soluble protein yield revealed only a weak correlation (*R*^2^ = 0.47) between fluorescence and activity (Figure 4A), while activity strongly aligned (*R*^2^ = 0.79) with soluble protein yield (Figure 4B). Fluorescence results may underestimate actual activity, as GFP fusion does not fully reflect the amount of soluble protein (Figure S2). A fluorescent protein may emit light even if the target protein is aggregated or misfolded, leading to fluorescence readings that do not necessarily correlate with soluble protein levels.^42^ Despite discrepancies between fluorescence and soluble protein yield, the fusion protein approach remains convenient for generating a quantitative estimate of recombinant protein levels. This allows for normalization and rapid calculations, providing a practical alternative to relying solely on reporter protein measurements for quantitative data.

### Define an Adjustment Index for TX-TL-FD Process

**Figure 5:**
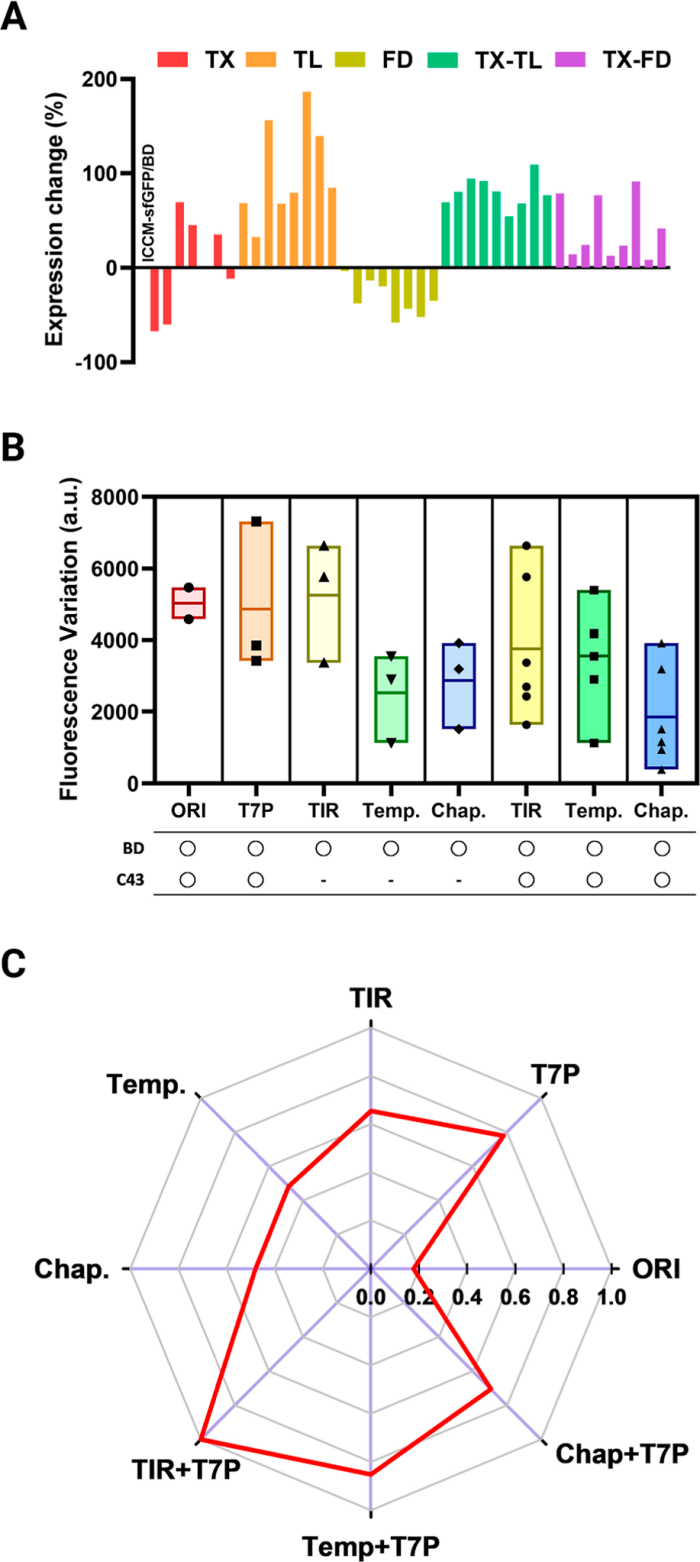
Integral analysis of regulating transcription (TX), translation (TL), and folding (FD) processes during protein production. (A) Relative changes in fluorescent signals from various engineered strains were normalized to the control strain pET28a-ICCM-sfGFP/BD. The data includes strains with modified 5′-UTR sequences, altered T7 RNA polymerase levels (e.g., C43(DE3)), and chaperone coexpression, as detailed in Table 2. (B) The distribution of fluorescent variation is derived from adjusting all regulating factors for protein production. (C) The adjustment index of regulating factors in the TX-TL-FD process. The variation of each regulatory factor was determined by the difference between its highest and lowest values. The relative significance was calculated by normalizing the difference between the highest and the lowest variation values.

The recombinant gene sequence follows a TX-TL-FD pathway to produce active proteins, and identifying key features within this process helps develop strategies to optimize protein expression. For challenging proteins like ICCM-sfGFP, precise regulatory adjustments within the TX-TL-FD process are crucial to enhance expression yield and stability, as shown by fluorescence comparisons (Figure 5A, Table S1). The result highlighted that transcription (TX) was the only factor influencing positive and negative relative expression changes in ICCM-sfGFP/BD (Figure 5A, red). The significant positive expression increases in translation (TL) regulation mainly stemmed from modifications to the recombinant sequence (Figure 5A, orange). Additionally, most adjustments in folding (FD) resulted in reduced expression levels (Figure 5A, yellow). TX-TL showed stable, specific-level expression improvement when combining TX with TL or FD regulation. At the same time, TX-FD adjustments shifted FD from adverse outcomes to positive (Figure 5A, green and purple). The critical regulatory factors in the TX-TL-FD remain unclear, highlighting the need for an additional parameter to assess expression changes for a more precise quantitative comparison.

The fluorescence variation (*V*-value) was the absolute change between the highest and lowest fluorescence results when adjusting individual regulatory factors. The *V*-value and range for each regulatory factor in the TX-TL-FD process were analyzed and compared (Figure 5B; Table 2). Results indicated that changes in T7RNAP level and TIR showed substantial variation in *V*-value, suggesting these factors are more responsive and diverse in their impact on expression. Furthermore, including expression data for TIR, temperature, and chaperone factors in C43 strains resulted in more extensive *V*-value ranges (Figure 5B). This finding emphasized that optimizing T7RNAP levels in the TX process significantly impacts recombinant expression and should be prioritized in optimization strategies.

**Table 2:** Specific Fluorescence, Variation Range, and Normalization Index Results from Different Hosts Involved in the TX-TL-FD Process

**hosts or conditions**	specific fluorescence (a.u./OD)							normalization index (0 to 1)
**host**	**ORI**			min	max	total range	variation range	
	BR322	SC101	UC					
BD	7608	2137	2598	2137	7608	5471	886	0.177
C43	11,032	9457	6447	6447	11,032	4585		
**Ori**	**T7RNAP level**							
	BD	ASIA	C43					
BR322	7608	8800	11,032	7608	11,032	3424	3896	0.779
SC101	2137	5758	9457	2137	9457	7320		
UC	2598		6447	2598	6447	3849		
**genes in BD**	**TIR**							
	T7S	T7B	T7S-LS					
ICCM	7608	10,977	8651	7608	10,977	3369	3270	0.654
hCAII	16,709	10,943	11,684	10,943	16,709	5766		
dCA	18,660	15,593	12,022	12,022	18,660	6638		
**host**	**temperature**							
	37 °C	30 °C	42 °C					
BD	7608	6304	4059	4059	7608	3549	2416	0.483
B7G	5638	5231	2734	2734	5638	2904		
BKJ	3694	3116	4249	3116	4249	1133		
**temperature**	**chaperone**							
	BD	B7G	BKJ					
30 °C	6304	5231	3116	3116	6304	3188	2399	0.480
37 °C	7608	5638	3694	3694	7608	3914		
42 °C	4059	2734	4249	2734	4249	1515		
**genes in different hosts**	**TIR**							
	T7S	T7B	T7S-LS					
ICCM/BD	7608	10,977	8651	7608	10,977	3369	5002	1.000
hCAII/BD	16,709	10,943	11,684	10,943	16,709	5766		
dCA/BD	18,660	15,593	12,022	12,022	18,660	6638		
ICCM/C43	11,032	11,755	12,668	11,032	12,668	1636		
hCAII/C43	12,504	11,788	10,077	10,077	12,504	2427		
dCA/C43	10,948	13,646	11,526	10,948	13,646	2697		
**hosts with different chaperone**	**temperature**							
	37 °C	30 °C	42 °C					
BD	7608	6304	4059	4059	7608	3549	4263	0.852
B7G	5638	5231	2734	2734	5638	2904		
BKJ	3694	3116	4249	3116	4249	1133		
C43	11,650	7458	8102	7458	11,650	4192		
C7G	11,521	7352	8063	7352	11,521	4169		
CKJ	12,466	7070	9224	7070	12,466	5396		
**temperature**	**host with chaperone**							
	BD	B7G	BKJ					
30 °C	6304	5231	3116	3116	6304	3188	3526	0.705
37 °C	7608	5638	3694	3694	7608	3914		
42 °C	4059	2734	4249	2734	4249	1515		
**temperature**	C43	C7G	CKJ					
30 °C	7458	7352	7070	7070	7458	388		
37 °C	11,650	11,521	12,466	11,521	12,466	945		
42 °C	8102	8063	9224	8063	9224	1161		

The relative significance should be clearly defined and quantified from the integral comparison of fluorescence variation. The “fluorescent variation window” for each regulatory group—the maximal variation range between the highest and lowest fluorescence values—was compared to the group with the highest range to evaluate the importance of regulating factors. These values were then normalized to produce an adjustment index (AI), resulting in a scale between 0 and 1 (Figure 5C). It highlighted T7RNAP regulation as the most impactful among the five individual regulating factors, reflected by its higher adjustment index (AI) values of 0.78. Additionally, adjusting the T7RNAP level and TIR simultaneously could generate the highest AI value and create more opportunities for optimizing the overproduction of hard-to-express protein in the TX-TL-FD process.

### New Insight into the Optimal Cutinase Production

Most easy-to-express proteins can be robustly expressed in BD strains without modifying T7 orthogonality or expression clusters. However, prioritizing regulatory factors in the TX-TL-FD process is vital for optimizing challenging proteins in *E. coli*. After pinpointing significant regulatory factors in the TX-TL-FD process, we conducted an in-depth analysis of ICCM to demonstrate further how adjustments to the interactions among transcription, translation, and folding influence the expression of challenging proteins.

**Figure 6:**
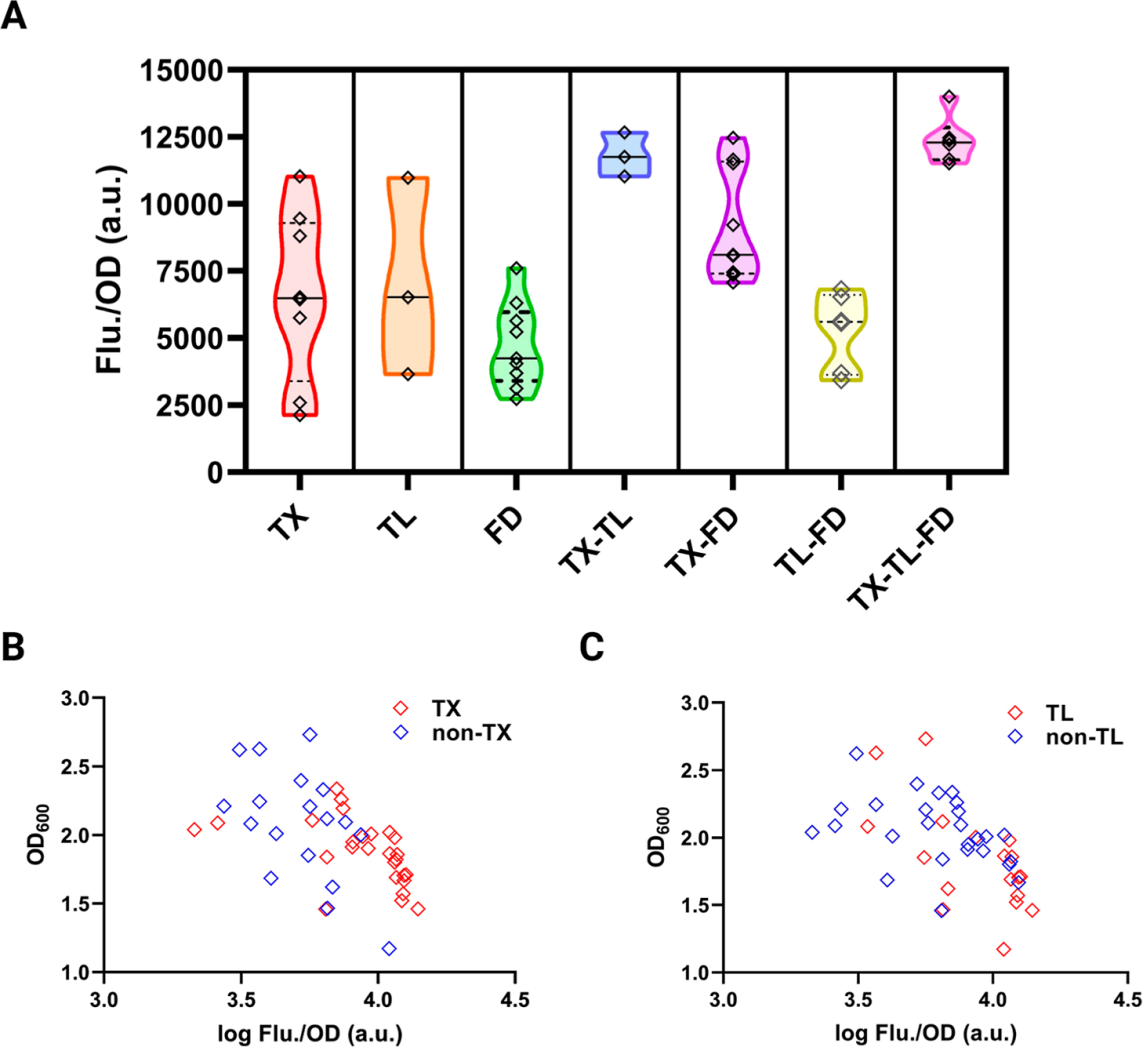
(A) The influence of regulating trisynergistic TX-TL-FD process on cutinase ICCM. The results include strains with modified 5′-UTR sequences, altering T7 RNA polymerase levels, i.e., C43(DE3) and chaperone coexpression as detailed in Table S2. (B) Distribution of TX-based (TX, TX-TL, TX-FD, TX-TL-FD) vs non-TX (TL, FD, TL-FD) and (C) distribution of TL-based (TL, TX-TL, TL-FD, TX-TL-FD) vs non-TL (TX, FD, TX-FD) effect for cell growth and specific fluorescence in terms of ICCM production in Figure 5A.

Based on the results in Table S1, Figure 6A showed the distribution of ICCM-sfGFP absolute fluorescence in every regulation combination of the TX-TL-FD process. Adjusting TX and TL significantly affected ICCM-sfGFP expression in *E. coli*, with fluorescence levels ranging from 2137 to 11,032 au/OD_600_, and combining TX with TL or FD further increased expression to 12,668 and 12,466 au/OD_600_, respectively (Figure 6A). Furthermore, it led to a more concentrated fluorescence distribution, with the minimum fluorescence boundaries for TX-TL and TX-FD combinations elevated to 11,032 and 7070 au/OD_600_, respectively. Although TL regulation created a significant difference in specific fluorescence, TL-FD regulation did not enhance expression levels or distribution boundaries as effectively as TX-TL or TX-FD regulation. Eventually, simultaneous regulation across all three categories in the TX-TL-FD process maximized ICCM-sfGFP expression, achieving the highest specific fluorescence of 14,007 observed in this study (Figure 6A).

For the challenging protein ICCM, it implied that the TX stage appeared to be the highest priority factor for optimization due to the significant variation in expression it produced. Moreover, regulation in TX-TL resulted in a positive variation of expression results corresponding to the relatively high adjustment index. Optimizing TX-TL proved the most effective for maximizing protein overproduction compared to other dual-factor strategies like TX-FD or TL-FD. This also demonstrated that the folding (FD) stage, supported by chaperones, positively contributes only after TX and TL have been sufficiently optimized, highlighting how folding efficiency is closely tied to the rate of amino acid synthesis.^43,44^ Ultimately, a cutinase ICCM fusion with sfGFP was maximized by coordinating factors across the TX-TL-FD pathway, achieving an 84% increase in protein yield compared to the direct use of BL21(DE3).

*E. coli* strains often face energy imbalances during overexpression with T7 systems, leading to low biomass yields, particularly when expressing challenging membrane proteins at low induction temperatures. Optimizing the TX-TL-FD process by regulating transcription factors often limited biomass to lower than 1 g/L, improving expression results (Figure 6B). Non-TX regulation results were scattered and showed a minimal correlation between biomass and fluorescence. Additionally, TL regulation can achieve high expression with lower biomass, while non-TL regulation showed more dispersion than non-TX regulation (Figure 6C). Unlike TX and TL, FD and non-FD regulation contributed to a wide distribution, with FD having a negligible impact on expression (Figure S3). The relationship between protein yield and biomass suggested that the energy trade-off between cell growth and T7 orthogonal expression is the primary reason transcription and translation regulation are critical in the TX-TL-FD process in BD strains. Previously, Lemo21(DE3) and Mutant56(DE3) were engineered *E. coli* strains optimized for expressing difficult-to-express proteins. Lemo21(DE3), with l-rhamnose-controlled expression, is particularly suited for membrane proteins, while Mutant56(DE3) benefits aggregation-prone proteins through enhanced folding capacity. In this study, the new chaperone-assisted strains provide alternative solutions to improve protein quality and yield. Moreover, integrating chaperones into different hosts may assist in expressing specialized proteins.

This framework identified T7RNAP levels and the translation initiation region (TIR) as pivotal elements. Coordinated regulation of transcription and translation (TX-TL) emerged as the most effective method for optimizing the expression of challenging proteins. Temperature adjustments significantly impacted expression efficiency more than chaperones, which were only effective when the TX and TL processes were balanced in *E. coli*. Impressively, the TX-TL optimization accounted for 90% of the improvement in harsh protein expression, with FD regulation contributing the remaining 10%. This highlights the critical factors in the TX-TL-FD pathway and the prioritization outlined by the adjustment index.

## Materials and Methods

### Integration of the Chaperone into the Chromosome

The P_T7_-groE and P_T7_-dnaKJ fragments were integrated into the *E. coli* genome using site-specific recombination, which required the CRIM plasmid and pAH69.^45^ First, the helper plasmid pAH69 was introduced into the host strain to enable integrase expression. Competent cells of the pAH69-harboring strain were prepared by growing the culture at 30 °C until an OD_600_ of 0.3 was reached, followed by a 30 min incubation at 39 °C to induce integrase production. The CRIM plasmid was introduced into the integrase-expressing host strain through heat shock transformation, followed by recovery at 37 °C for 3 h. Positive colonies were then screened on LB agar containing 25 μg/mL kanamycin at 37 °C to prevent the helper plasmid’s loss. A marker-free strain was generated using the pCP20 plasmid containing a heat-inducible FLP recombinase to excise the FRT-flanked resistance gene. Single colonies of pCP20-harboring strains were randomly selected and precultured in LB with ampicillin at 30 °C. The cells were grown in LB broth without antibiotics at 37 °C for 6–8 h, then shifted to 39 °C for 1 h. After incubation on an LB plate at 37 °C overnight, single colonies were picked and screened on kanamycin and ampicillin plates. Moreover, colony PCR was used to confirm the fragment from the T7 promoter to the open reading frame, as shown in the maps in Figure S4. Finally, B7G, BKJ, C43(DE3), C7G, and CKJ were generated.

### Plasmids Construction

The plasmids and strains are summarized in Table S2. The vectors with pUC and pSC101* *ori* were digested by *Xho*I and *Pst*I, and the PCR fragments were inserted to generate the plasmids of pSUI-T7-sfGFP and pSCKI-T7-sfGFP, respectively. The ICCM cutinase sequence, derived from a mutant of the original leaf compost cutinase, was synthesized by Integrated DNA Technologies (IDT). Human carbonic anhydrase II (hCAII) and the *de novo* enzyme dCA12^46^ were used for comparison. The fusion protein ICCM-sfGFP was amplified via PCR and digested by *Nde*I and *Xho*I to obtain the pET28a-ICCM-sfGFP, pSUI-ICCM-sfGFP, and pSCKI-ICCM-sfGFP. For hCAII-sfGFP and dCA12-sfGFP, the PCR products were digested by *Nde*I/*Xho*I and inserted into pET28-sfGFP to construct pET28a-hCAII-sfGFP and pET28a-dCA12-sfGFP. Finally, the RBS of B0034 and the leader sequence (LS) were replaced or inserted into pET28a-ICCM-sfGFP, pET28a-hCAII-sfGFP, and pET28a-dCA12-sfGFP to test the translation efficiency. All new vectors after construction were transformed into various expression hosts.

### Culture Conditions

The different recombinant *E. coli* were first grown overnight on LB plates containing the appropriate antibiotics (50 μg/mL kanamycin or 25 μg/mL chloramphenicol). A single colony was precultured in 4 mL of LB medium at 30 or 37, or 42 °C with shaking at 180 rpm for 16 h. The culture with 20 mL of LB in the flask was operated under the same conditions, and measured OD_600_ using a SpectraMax M2 spectrophotometer (Molecular Device, USA). When the OD_600_ reached 0.6, the cells were induced with 0.1 mM IPTG. After 24 h, cells were harvested by centrifugation at 10,000*g* for 5 min at 4 °C, washed twice with deionized water, and adjusted to an OD_600_ of 4. The cells were then disrupted using a high-pressure homogenizer (OneShot, UK), followed by centrifugation at 13,000*g* for 10 min at 4 °C to collect the supernatant for activity testing.

### Fluorescent Analysis

The cells were cultured in LB medium under specific conditions for 12 h, and 0.2 mL was transferred to a 96-well plate. Cell density was assessed using optical density (OD) at 600 nm, whereas the fluorescence of sfGFP was quantified with excitation at 480 nm and emission at 510 nm using SpectraMax M2 (Molecular Devices, USA).

### *p*-Nitrophenyl Butyrate (*p*-NPB) Assay for ICCM Activity

The cutinase ICCM activity was assessed using *p*-NPB (Sigma-Aldrich N9876) as substrate with specific adjustments. A 1 mM *p*-NPB in pure dimethyl sulfoxide was mixed with the enzyme in 25 mM Na_2_HPO_3_–HCl buffer at pH 7. The crude protein concentration was adjusted to an OD600 of 4 for apparent activity analysis. The formation rate of *p*-NPB hydrolysis products was measured over 10 min at 400 nm using a spectrophotometer (Molecular Devices, USA). An enzyme activity was defined as 1 μmol of p-nitrophenol produced per minute.

### SDS-PAGE Analysis for Protein Amounts and Pattern

The cells were washed twice with deionized water and concentrated to an OD_600_ of 4. Whole-cell and soluble protein samples were mixed with protein dye and heated at 100 °C for 5 min. SDS-PAGE was then performed with a 12% separating gel and a 4% stacking gel. Proteins were stained with Coomassie blue R-250. Finally, the gels were scanned with a Bio-5000 Plus image scanner (Microtek, Taiwan). Protein quantification was conducted using ImageJ software, with the highest expression level set to 100%.

### Transcriptional Level Analysis

Total RNA was extracted from cells cultured during the exponential phase (i.e., 6 h after induction) using the Total RNA Isolation Kit (GeneDirex, Germany). Complementary DNA (cDNA) synthesis was done using 1 μg of total RNA with a cDNA Synthesis Kit (LIGHT Biotech, Taiwan). Quantitative PCR was performed on the StepOne Real-Time PCR (Applied Biosystem, USA) using the EvaGreen Master Mix (Blossom Bio, Taiwan). Gene expression levels, relative to 16S rRNA, were calculated by the 2^–ΔΔCT^.

### Plasmid Copy Number Determination

The plasmid copy number (PCN) of strains was measured after postinduction at 6 h. Bacterial cells were collected by centrifugation, washed twice with deionized water, and resuspended in deionized water. The suspension was heated at 95 °C for 10 min. After centrifugation, the supernatant was used for qPCR analysis with the EvaGreen qPCR System-ROX I (GeneDireX, Germany) on a StepOnePlus Real-Time PCR System (Applied Biosystems, USA). The assay targeted the kanamycin resistance (Km) gene to determine the plasmid copy number (PCN), using the Can gene as the single-copy reference. PCN was calculated with the 2^–ΔCt^ formula, where ΔCt is the difference in Ct values between Km and *can* gene in *E. coli*.

## Supporting Information Available

Supporting Figure (S1) SDS-PAGE analysis of the leader sequence (LS) on different proteins, (S2) SDS-PAGE analysis of ICCM-sfGFP expression in different hosts, (S3) distribution of FD-based (FD, TL-FD, TX-TL-FD) vs non-FD (TX, TL, TX-TL), and (S4) maps of the plasmid for the engineered chaperone on the chromosome of *E. coli*. Supporting Table (S1) fluorescence signal of ICCM-sfGFP under different conditions in the TX-TL-FD process, and (S2) detailing of strains and plasmids (PDF)
