# Supplementary material for: Deciphering Transcription-Translation-Folding (TX-TL-FD) for Enhancing Cutinase Production in T7 System and Genetic Chaperone-Equipped Escherichia coli Strains
Source: ACS Synth Biol. 2025 May 7;14(5):1843–52. doi: 10.1021/acssynbio.5c00245 (PMC12090337; doi:10.1021/acssynbio.5c00245)
Supplement: Supplementary file 1 — sb5c00245_si_001.pdf [file sb5c00245_si_001.pdf]

Supporting information

Deciphering Transcription-Translation-Folding (TX-TL-FD) for Enhancing Cutinase Production in T7 system and Genetic Chaperone-equipped *Escherichia coli* Strains

Chuan-Chieh Hsiang, I-Son Ng*

Department of Chemical Engineering, National Cheng Kung University, Tainan 701, Taiwan

*Corresponding author: Prof. I-Son Ng

Tel: +886-62757575-62648; Fax: +886-62344496

E-mail: yswu@mail.ncku.edu.tw

ORCID: 0000-0003-1659-5814

Table S1. The fluorescence signal of ICCM-sfGFP under different regulating controls in the TX-TL-FD process.

**Process and strains**	Fluorescence of ICCM-sfGFP (a.u.)		
**TX**	**Replication origin**		
	pBR322	pSC101	pUC
BD	7608	2137	2598
ASIA	8800	5758	N.D.
C43	11032	9457	6447
**TL**	**TIR**		
	T7S	B0034	T7S-LS
ICCM/BD	7608	10977	8651
**FD**	**Temperature (°C)**		
	37	30	42
BD	7608	6304	4059
B7G	5638	5231	2734
BKJ	3694	3116	4249
**TX-TL**	**TIR**		
	T7S	B0034	T7S-LS
ICCM/C43	11032	11755	12668
**TX-FD**	**Temperature (°C)**		
	37	30	42
C43	11650	7458	8102
C7G	11521	7352	8063
CKJ	12466	7070	9224
**TL-FD**	**TIR**		
	T7S	B0034	T7S-LS
ICCM/B7G	5638	5567	6815
ICCM/BKJ	3694	3429	6537
**TX-TL-FD**	**TIR**		
	T7S	B0034	T7S-LS
ICCM/C7G	11521	11674	12217
ICCM/CKJ	12466	12373	14007

Table S2. Strains and plasmids used in this study.

Strains and plasmids	Description
Strains	
DH5α	F− φ80lacZΔM15 Δ(*lac*ZYA-*arg*F)U169 *rec*A1 *end*A1 hsdR17 (rK −, mK +) *pho*A *sup*E44 λ⁻ *thi*-1 *gyr*A96 *rel*A1
BL21(DE3)	F– *omp*T *hsd*SB ($r_B{}^-$, $m_B{}^-$) *gal dcm* (DE3)
C43(DE3)	Derived from C41(DE3)
B7G	BL21(DE3) integrating P_{T7}-*gro*E cluster
BKJ	BL21(DE3) integrating P_{T7}-*dna*K-*dna*J cluster
C7G	C43(DE3) integrating P_{T7}-*gro*E cluster
CKJ	C43(DE3) integrating P_{T7}-*dna*K-*dna*J cluster
$ASIA^{hsp}$	BL21(DE3) harboring pSAC-P_{hsp}-LysY
Plasmids	
pHK-T7-GroELS	R6K ori, T7 promoter, GroELS, HK attP, FRT site, Km^R
pHK-T7-DnaKJ	R6K ori, T7 promoter, DnaKJ, HK attP, FRT site, Km^R
pET28a-sfGFP	pBR322 ori, lacI, T7 promoter, sfGFP, T7 terminator, Km^R
pSCKI-T7-sfGFP	pSC101 ori, lacI, T7 promoter, sfGFP, Km^R
pSUI-T7-sfGFP	pUC ori, lacI, T7 promoter, sfGFP, Km^R
pET28a-ICCM-sfGFP	pBR322 ori, lacI, T7 promoter, ICCM fused with sfGFP, T7 terminator, Km^R
pSCKI-ICCM-sfGFP	pSC101 ori, lacI, T7 promoter, ICCM fused with sfGFP, Km^R
pSUI-ICCM-sfGFP	pUC ori, lacI, T7 promoter, ICCM fused with sfGFP, Km^R
pET28a-hCAII-sfGFP	pBR322 ori, lacI, T7 promoter, hCAII fused with sfGFP, T7 terminator, Km^R
pET28a-dCA12-sfGFP	pBR322 ori, lacI, T7 promoter, dCA12 fused with sfGFP, T7 terminator, Km^R
pET28a-B34-ICCM-sfGFP	pBR322 ori, lacI, T7 promoter, B0034 RBS, ICCM fused with sfGFP, Km^R
pET28a-LS-ICCM-sfGFP	pBR322 ori, lacI, T7 promoter, leader sequence (LS), ICCM fused with sfGFP, Km^R
pET28a-B34-hCAII-sfGFP	pBR322 ori, lacI, T7 promoter, B0034 RBS, hCAII fused with sfGFP, Km^R
pET28a-LS-hCAII-sfGFP	pBR322 ori, lacI, T7 promoter, leader sequence (LS), hCAII fused with sfGFP, Km^R
pET28a-B34-dCA12-sfGFP	pBR322 ori, lacI, T7 promoter, B0034 RBS, dCA12 fused with sfGFP, Km^R
pET28a-LS-dCA12-sfGFP	pBR322 ori, lacI, T7 promoter, leader sequence (LS), dCA12 fused with sfGFP, Km^R
pSAC-P_{hsp}-LysY	p15A ori, P_{hsp}, B0034 RBS, LysY, Cm^R

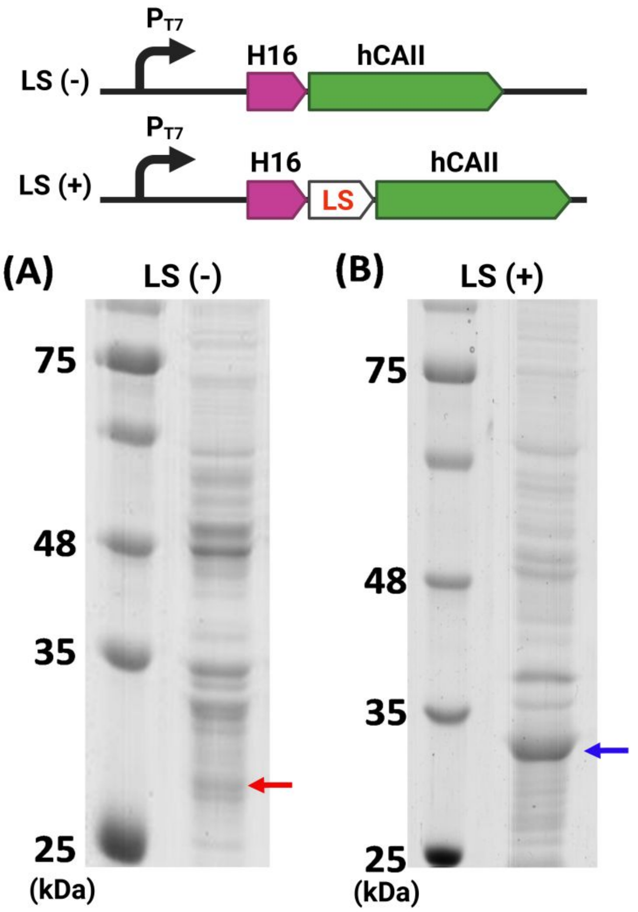

Figure S1. SDS-PAGE analysis illustrating the rescuing effect of the leader sequence (LS) on the expression of a challenging protein. In the LS(-) design, the H16 peptide is directly fused to the N-terminus of hCAII, whereas in the LS(+), the leader sequence is included after H16 peptide and fused to hCAII. Both constructs were expressed in C43(DE3). (A) hCAII is indicated by a red arrow and (B) by a blue arrow. The cells were induced by 0.1 mM IPTG and cultured at 37℃.

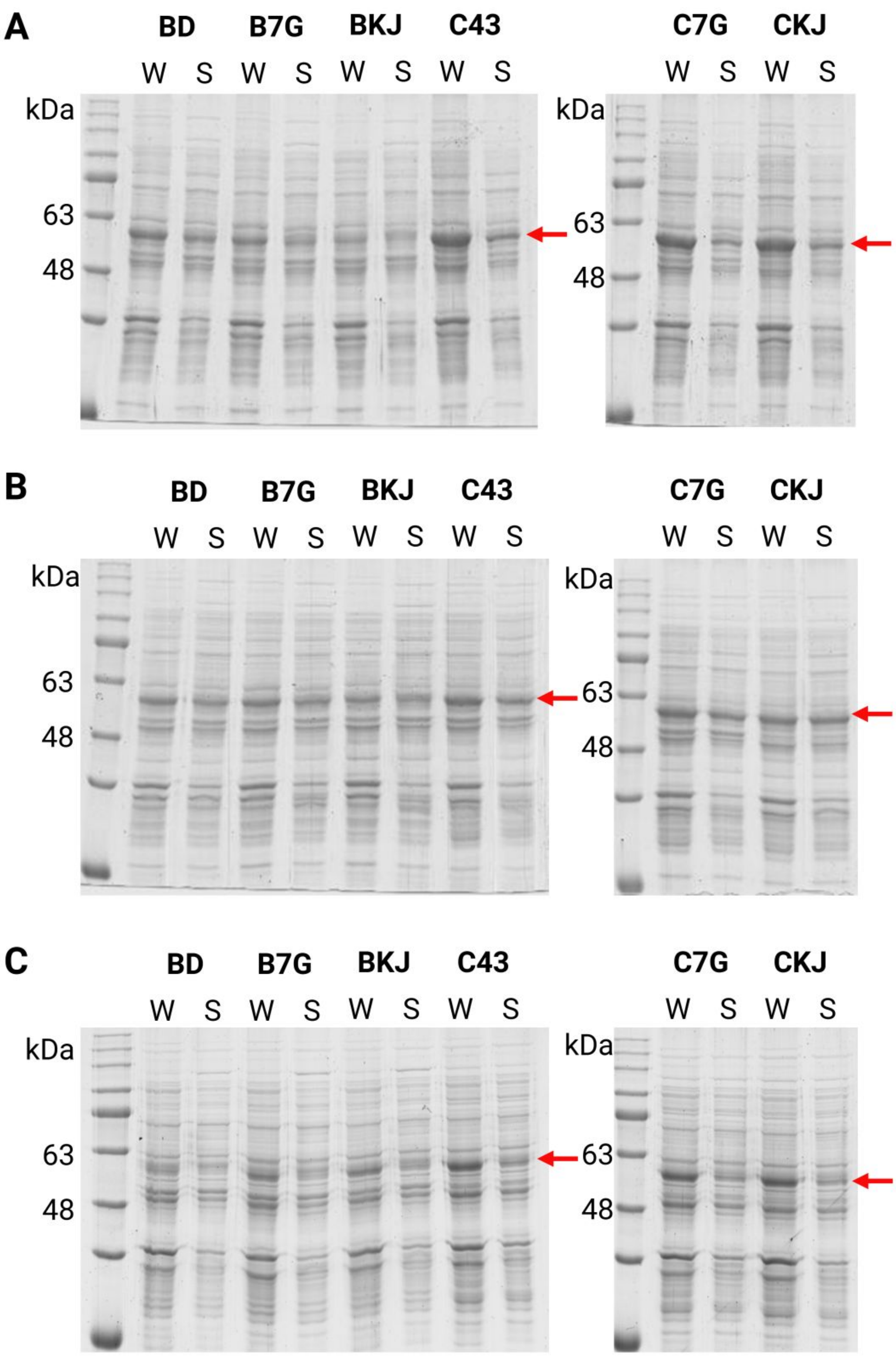

Figure S2. SDS-PAGE analysis of ICCM-sfGFP expression in BL21(DE3), C43(DE3), and engineered strains B7G, BKJ, C7G, and BKJ under induction at (A) 37°C, (B) 30°C, and (C) 42°C. The strains were induced by 0.1 mM IPTG. Red arrows indicated the target protein.

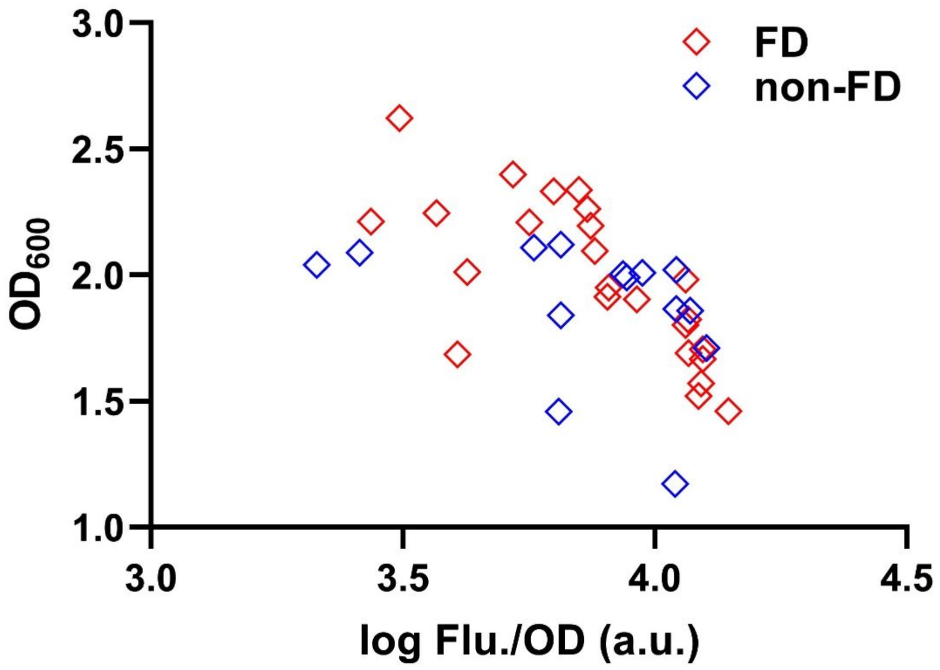

Figure S3. Distribution of FD-based (FD, TL-FD, TX-TL-FD) v.s. non-FD (TX, TL, TX-TL) effect for cell growth and specific fluorescence in terms of ICCM production in Figure 6A.

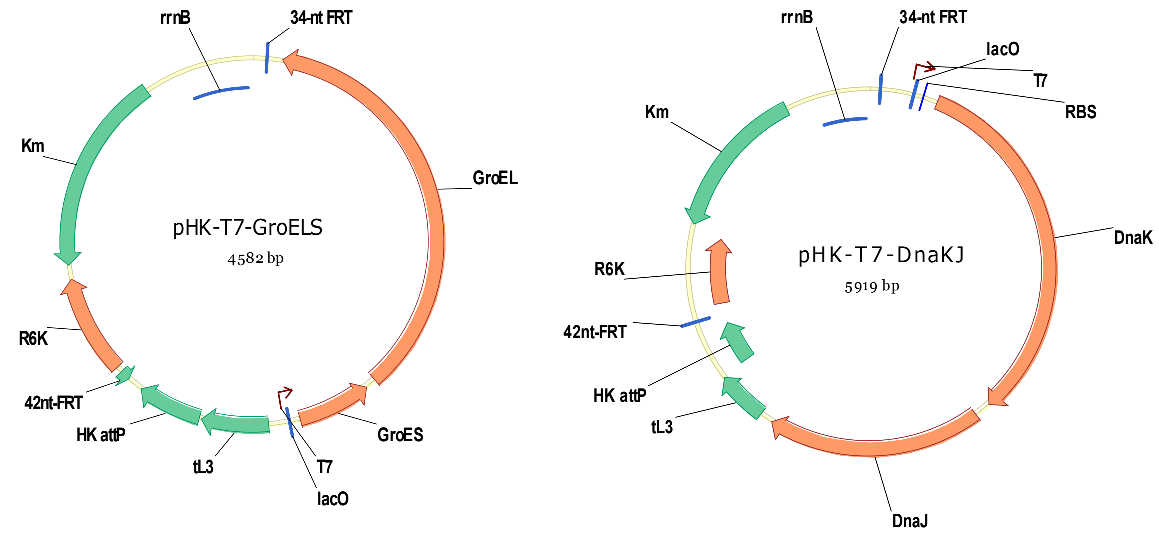

Figure S4. Genetic map of pHK-T7-GroELS and pHK-T7-DnaKJ for integration the chaperones to HK022 phage attack site on *E. coli* chromosome.
